# Can Functionalised Play Make Children Happy? A Critical Sociology Perspective

**DOI:** 10.3389/fpubh.2020.571054

**Published:** 2020-09-24

**Authors:** Annika Frahsa, Ansgar Thiel

**Affiliations:** ^1^Institute of Sport Science, University of Tüebingen, Tuebingen, Germany; ^2^Interfaculty Research Institute for Sport and Physical Activity, University of Tüebingen, Tuebingen, Germany

**Keywords:** children's play, individualization, functionalization, digitalization, child health, medicalisation, physical activity monitoring

## Abstract

The functionalisation of play basically stems from the diagnosis of a global childhood inactivity crisis, the so-called “children's obesity pandemic.” Hence, in the context of the activity-related guidelines, children's play appears no longer to be unproductive and purpose-free. It is rather considered an anti-obesity tool that will help children to meet physical activity recommendations. It is questionable whether such a functionalised tool can also provide what has been called the salience of the pleasures of free-play for children. Furthermore, a “normalization” of functionalised practices of play, in turn, could stigmatize children who do not or cannot adhere to these practices. Based upon this background, this paper will take a critical sociology perspective to analyse the functionalisation and medicalisation of children's play in an individualized, mediatized, and pedagogised society. In this sense, the paper aims to pay attention to how the primary goal of play in the sense of “simply make children happy” has given way to the goal of making them healthy and functional.

## Introduction

Play has been perceived as so important to optimal child development that it has been recognized by the United Nations High Commission for Human Rights. According to the UN Convention on the Rights of the Child, every child has the right “to rest and leisure, to engage in play and recreational activities” (1). At the same time, current research reports a decline in play among children in the Global North, particularly for middle-class, female, younger, and ethnic-minority children (2).

Historical analyses have highlighted that childhood play is a natural human trait, but its extent depends on socio-cultural conditions in which people live. During many historical eras, such as post-hunter-gatherer societies, in the ages of agriculturalization, or following the industrial revolution, children had only limited opportunities to play because they had to spend most of the daytime working (3). Playing nevertheless occurred, whenever possible, sometime intertwined with labor, without parental or generally adult supervision and direction. Children's play tended to be free and spontaneous; in rural regions it took place in fields, streams, and farmyards, in urban regions it tended to be located in vacant lands and parks (4). However, it was not until the Age of Enlightenment, with the discovery of childhood as an independent phase of life, that children's play was recognized as a human developmental necessity (5).

Children's play, as we know it today, is a phenomenon of modern society. Authors such as Chudacoff (4) have identified the mid-1950s as “golden age” of children's play in North America and potentially the Global North in general. After World War II, children tended to be free from long working hours. Generally, children's play was positively attributed, which was also reflected in the development of supportive environments, such as parks and other playgrounds. However, this “golden age” did not last long. Starting already in the mid 1950s, children's play has been continuously declining. Part of this decline has been attributed to the role of adults and their control and supervision of children's activities (3).

In the following, we will take up the challenge of understanding and explaining this decline and its consequences for public health. Understanding and explaining changes in play requires a broader perspective. Thus, we will apply a critical sociological lens to analyze occurring changes in play. We follow an understanding of children's play as *free play*, as an “activity that is freely chosen and directed by the participants and undertaken for its own sake, not consciously pursued to achieve ends that are distinct from the activity itself” (3). Our central assumption is that free play of children has been increasingly replaced by playful activities which are functionalized in the sense that they are aimed at fulfilling a purpose that is outside the activity. Hence, we argue against the background of the notion that children's leisure lives are reframed (6) in the sense that the basic intention of play to “simply make children happy” (7) has given way to the goal of making them healthy and functional.

In building our argument, we will first provide a critical reflection about four recent trends in today's society that have had an impact on children's play: (i) individualization, pedagogisation, and protection, (ii) indoor- and setting-orientation (iii) mediatisation and digitalization, as well as (iv) medicalisation. Following the critical reflection of those trends, we will discuss the potential consequences of functionalisation to the pleasures linked to free play. We will conclude the paper by arguing for a more holistic understanding of free play to be integrated into physical activity promotion and monitoring in public health.

## Trends in Children's Play

### Individualization, Pedagogisation, and Protection

In principle, modern meritocratic societies are characterized by the fact that social advancement is not *per se* predetermined by social background. However, the chances of professional success are—among other factors—limited by qualifications and skills, which have to be developed. In this sense, children find themselves in a “state of permanent endangerment” (8). This has consequences, both at the micro- and at the macro-level (9). At the macro-level, the development of talents in each individual has become a central goal of education policy. Hence, the design of learning environments has increasingly focused on a methodically and didactically professionalized promotion of individual strengths and self-development (10). We observe that this pedagogical aspiration has been extended to the earliest childhood, for example, in foreign language courses for 3-months-old babies or special toys for highly gifted toddlers. We call this a “pedagogisation” that also impacts the way in which a child's social sphere is organized at the micro-level. Children's life worlds have become “pedagogised” in order to limit the risks of uncertain biographies and to offer children and adolescents the best possible start in life.

One consequence is that performance pressure already starts in early childhood. The pedagogisation of children's life worlds also causes so-called “helicopter parents” to hovering over their children, eagerly trying to support them wherever they can in order to solve their problems and promote their success (11, 12).

With regard to the spatial structure of these life-worlds, childhood in today's societies tends to be characterized by functionalized “islands.” Children and adolescents commute between separate islands throughout the day, from daycare or school to selected enrichment activities, including individual and collective extracurricular sporting, cultural, and leisure opportunities (2). This island structure of the children's life worlds makes children's life dependent on continous parental support. If parents want to provide their children with the broadest possible support for development, they have to transport them from one island to another: from school to the sports club, to the music school, and even to the playdate with friends. Consequently, parents take control over both children's everyday and leisure-time activities as well as their everyday and leisure-time mobility.

As a result of these societal processes of individualization and pedagogisation, time and significance given to schooling and other adult-directed school-like or enrichment activities have increased (3, 13). Increases in time spent at school, pre-school, and kindergarten have come along with decreases in break times. Play tends to take place within structured settings such as P.E. and supervised breaks, daycare and after school care in clubs or recreation centers. Due to the fact that these scheduled activities do not necessarily provide links between each other, children mostly do not have relationships to other children that go beyond the functionalized islands. Furthermore, the pedagogisation of children's lifestyles considerably restricts the opportunities to autonomously explore social spaces in between the specialized islands. Consequently, the time children spend play freely has continuously decreased over the last decades. Data indicates that the time children spent in free play decreased by about 25% in the USA, already between the 1980s and late 1990s (14). Instead, children's games have become functionalised. To promote children's development in the best possible way, they serve a predetermined purpose, take place at a fixed location, and are instructed by professionals (15).

At the same time, parent-children interaction with regard to children's play has changed. For the 1990s, Valentine and McKendrick showed that, due to adult restrictions and parental rules, children's freedom declined significantly for outdoor play and travel in their neighborhoods (16, 17). Several studies have provided more insights on parenting styles to free play, particularly free unsupervised outdoor play. Parenting styles have become of interest to popular media as well as research, and multiple hyper-parenting styles have been identified (18). The already mentioned “helicopter parents” tend to be overly involved and protective parents who supervise their children's activities and often intervene in their children's affairs and make decisions for them (12, 19, 20). Janssen (18) referred to further parental styles, introduced by other authors, that hinder children's autonomy: “Little emperor” parents focus on providing all material goods available to and craved by children. “Tiger moms” strive for extraordinary achievement among their children. Finally, parents characterized by “concerted cultivation” provide children with a plethora of extracurricular activities aimed at giving the children socio-cultural advantages compared to their peers.

Hyper-parenting styles have influenced children's outdoor play (21). Studies showed that more fear for children's safety is linked to less free play (21–25). In this regard, the desire to prevent potentially risky situations is a main barrier toward the exploration of unknown spaces by the children. Consequently, games that had been perceived as normal in earlier generations are now considered dangerous (26). Fear and danger might either lead parents to eliminate children's play, such as to completely forbid unsupervised outdoor play, or to modify play by putting it under parental supervision, accompanying children in every while playful activity or limiting play to the own backyard (27).

### Mediatisation and Digitalization

The consequences of individualization and pedagogisation for children's play are amplified by mediatisation and digitalization. It is not only that parents can use media and digital gadgets to constantly supervise their children's play and their locations (28). In today's Global North, the children's world itself is shaped by information and communication technologies. Due to the digitalization of life-worlds, socio-spatial conditions of growing up have fundamentally changed. Internet-based communication technologies have become a natural part of everyday life. According to Statista, almost every adolescent over 11 owns a smartphone in Germany (29). For some adolescents, smartphones have become so important for coping with everyday life that they perceive these devices as a parts of their own body (30).

With regard to the structures of everyday communication, instant messaging (e.g., WhatsApp, Snapchat, iMessage, or Hangout) has become much more important for the communication with peers than phone calls (31), potentially also more important than face-to-face communication. Digitalization boosts the individualization of everyday communication (9). Communication via WhatsApp or Snapchat does not rely on instantly responding to messages, as it is the case with analog face-to-face communication. Messages are read and answered, when it suits the recipients. Digitalization also impacts social relationships. Emotions are expressed by emoticons and emojis. Facial expressions and nonverbal gestures, fundamentally important for analog communication, play a minor role in digitalized communication.

In a digitalized society, adolescents do not communicate less. However, it is different *how* they communicate. Social negotiation processes play a less important role. In many cases, it is no longer important to “read” body language. The social restrictions of digitalized everyday communication may lead to deficits in adolescents' development of social skills which are relevant for playing games in analog worlds (32). The shift toward digital activities might also minimize positive effects associated with offline events, such as opportunities to increase social capital, build and enlarge networks as well as transform relationships to a deeper level (33).

On the other hand, the internet enables us to transcend concrete geographic space. The ability to communicate virtually independent of time of day and location provides the opportunity to create virtual “meeting places” (34). In this sense, digitalized communication offers participation and inclusion in an otherwise fragmented lifeworld by creating a second “virtual” reality, including new playgrounds and meeting places. At the same time, certain multiplayer online role-playing games might allow for digital social interaction and positive effects on social capital (35). Those might result in new friendships that can deepen through shared interest whereas non-playing offline friends might get neglected.

The trend toward digitalization will ultimately result in a, at least partly, transfer of play into virtual playfields or arenas. Especially virtual playfields are nowadays increasingly used to promote physical activity among children.

### Medicalisation

One consequence of individualization and mediatisation of children's play is the trend toward creating games for children to serve health-related purposes. Behind the development of exergames or serious games is the idea that children who tend to be inactive can be motivated to exercise via playful and enjoyable activities. The WHO, for example, states that “for children who are currently inactive, progressive increase in activity to reach the target through additional time for free active play will have health benefits” (36). With similar intent, a clinical report by the Committee on Psychosocial Aspects of Child and Family Health and the Council on Communications and Media of the American Academy of Pediatrics (37) emphasizes the health-related benefits of play even aims aim to provide information “to write a prescription for play at well visits to complement reach out and read” (37).

This “medicalization of play” basically stems from the diagnosis of a global childhood inactivity crisis, the so-called “children's obesity pandemic.” Hence, in the context of the activity-related guidelines, children's play is primarily discussed to tackle the problem of inactivity (38–40). In this regard, the Active Healthy Kids Global Alliance developed a matrix for national report cards on children's core physical activity, initiated by Active Healthy Kids Canada in 2004 (39). The matrix considers “active play” as one indicator of core physical activity (41). So far, Active Healthy Kids research working groups from 49 countries have adopted the procedure and presented national report cards. For Germany, Demetriou et al. (38) have very recently presented the German report card and concluded that <25% of children and adolescents play actively for several hours per day.

The flipside of this well-meant trend is that children's play no longer appears to be unproductive and purpose-free. It is rather considered an anti-obesity tool that will help children reach the recommended 60 min of physical activity per day (36). Data already has indicated that functionalised play could become a privileged and exclusive support activity for some children only. Middle-class children in England participate to a higher degree in functionalised play activities, such as extracurricular sporting and leisure activities, than working-class children, even though parents of both classes value such activities in similar ways (2). A relevant factor in this regard is the ability to incorporate functionalised play into the life-worlds of families. Since this transfer seems to work better in middle-class families, functionalised playing activities in these families can compensate the loss of free unstructured play to a higher degree than working-class families in England (2). Hence, a “normalization” of functionalised practices of play could stigmatize children who do not or cannot adhere to these practices (42).

Furthermore, it is questionable whether a functionalised anti-obesity tool can also provide what Sutton-Smith has called the salience of the pleasures of free play for children (7).

## Can Functionalized Play Provide the Pleasures of Children's Free Play?

Data already indicates that the restriction of free play may have a negative impact on the general well-being of children (43, 44). Free play is an activity that is (a) voluntary, (b) open and uncertain, (c) separated from everyday life, (d) unproductive and self-sufficient, (e) regulated, and (f) fictional (45).

First, *voluntary participation* is indispensable for play, one cannot really be forced to play. Play only takes place “when the players have a desire to play, and play the most absorbing, exhausting game in order to find diversion, escape from responsibility and routine” (45). Play thus represents a space of action sought for its own sake. Play enables the actors to satisfy their current needs for action and to experience the joy of immediate activity. Play is an activity that is motivated by the quality of life, excitement, satisfaction, and joy experienced in the course of action. People can become absorbed in play, fully immerse themselves in the activity, receive intensive feedback about their own abilities, find a pleasure-oriented approach to (physical) culture and thereby experience a feeling of initiative and causation through play. Second, the *openness and uncertainty* of the outcome is a further characteristic of play. Particularly for children and adolescents, the uncertainty—which is often difficult to bear—and the associated risk of failure seem to be dimensions of play that promote individual development and growth. Playing confronts children and adolescents with multiple challenges and it supports to establish a relational relationship between someone's world of experience and the person's relevant environment. While playing, children exhibit a multitude of social behaviors and interactions, and take up various social status roles (46). These roles mirror to a certain extent the life-worlds, in which the children are growing up. Third, play is *a separate activity* that takes place in a special world that is clearly distinguishable from the world of day-to-day life. Play takes place in its own time and space. Ideally, nothing is created through playful activity that is of significance outside that activity. In this sense, play is, fourth, an *unproductive activity* that is sufficient in itself but in contrast to the ordinary day-to-day life. Fifth, since cultural ideas and conventions have an effect on how to play, play appears to be a *regulated activity*, but its rules may well conflict with the legal or day-to-day regulations of the living environment. Due to the conscious “as-if” character of the activity, play is, sixth, also a *fictional activity* whose “unreality” stimulates dedicated playing and the experience of joy both during as well as after play.

In principle, children's play can contribute to the development of intrinsic motivation and competence, decision-making and problem-solving skills, self-control and emotional regulations, friendship skills and social capital (3). Children's free play also embodies the dynamic of the dramatic increase and decrease of tension. Hence, play allows to practice the drama of interpersonal relationships in a protected environment in a casual way (47). As such, play plays an important role in Piaget's process of accommodation and assimilation and also serves for empowerment.

The fact that children's play particularly contributes to the experience of joy makes it such a valuable tool for public health programs. From the perspective of critical sociology, however, it is questionable, whether activities like “energetic play” (36), “free active play” (36), “active play” (38, 41) or “active outdoor play” (39, 48), which are implemented as parts of public health programs, are indeed fun for the children. The use of these terms in the context of public health is not neutral. Rather, it can be regarded as a sign of objectification and functionalisation in the current discourse on play in public health and physical activity promotion (26). Making “active play” or “active free play” a research object and promoting it as part of public health concerns may (re-)define the way play is understood and experienced. From the perspective of a sociology of the child, Alexander et al., among others, have already hinted at the underlying paradox in this discourse: advocating, promoting and discussing children's play might reshape it into a new form that is purpose-driven and thus no longer free and intrinsically motivated (6). In this sense, a highly pre-structured, functionalised playing activity bears the risk that it no longer primarily aims at what Sutton-Smith identifies as the main function of free play of children: that it simply “makes them happier” (7).

From the perspective of critical sport sociology, a more reflective discussion of functionalized play is needed that pays attention to the current “reframing of children's leisure lives” (6). Such a critical reflection should particularly focus on the potential harmful consequences of transforming a leisure activity that allows risk-taking and provides fun (which is what childhood should be about in the first place) into a much more constrained, managed, and supervised intervention.

The functionalisation of play in the field of health promotion has a particularly interesting dynamic in the form of so-called “serious games.” Marsh (49) defines serious games as follows: “*Serious games are digital games, simulations, virtual environments and mixed reality/media that provide opportunities to engage in activities through responsive narrative/story, gameplay or encoun- ters to inform, influence, for well-being, and/or experience to convey meaning. (…) Serious games are identified along a continuum from games for purpose at one end, through to experiential environments with minimal or no gaming characteristics for experience at the other end.”* These games are developed, designed, marketed and distributed by companies. Within the framework of the serious games, health-related content is taken up and integrated into an exciting story, which should ensure a sustainable motivation for healthy behavior. At the same time, the companies are oriented toward selling the games and thus primarily follow a market logic. To the extent that business enterprises, due to their market interest, integrate algorithms into the games that create dependency on playing or even play addiction, the health promotion intention is thwarted. Thus, e-games may deviate from the idea of free children's games in two respects: On the one hand, the games are pre-programmed, methodised and internationalized to a large extent. In this regard, one can also expect effects of social inequality. In milieus, in which physical activity is considered a relevant instrument to foster the development of children, functionalised e-games will probably be given to children, while milieus that are far away from movement will become impoverished in terms of physical activity. On the other hand, the attempt to encourage children to consume these games in a sustainable way and—linked to this—to buy updates or new versions, prevent adolescents from freely deciding for or against the game. Given that the particularly motivating health-promoting e-games for children are most at risk to cause dependency, guidance of children becomes of central importance.

Current statistics on the media behavior of children during the COVID-19-pandemic show how important supervision of children's use of digital technologies is. According to a recent German FORSA survey with 500 children on behalf of a large German health insurance, 95 percent of all parents report that their children spent significantly more time in front of PCs and smartphones during time of school lockdowns (50). In this regard, it is not only the use of communication technologies, social media, streaming services or e-games, but also the massive upswing of digital learning that leads to an increase of time spent sedentarily. Only in a small percentage of the children, this behavioral change is compensated by physical activity, for example by more outdoor activities in gardens or parks (38 percent). A Facebook Messenger survey on behalf of ParentsTogether with over 3,000 parents came to a similar conclusion in the USA (51). The average time spent online has doubled for kids during the crisis. Almost half of the children spent more than 6 h online per day (compared to only 8.29% before), 26% of kids even spent more than 8 h online (compared to 4% previously) during the lockdown. The most prominent platforms and apps used by children whose parents completed the survey were mostly non-educational: YouTube (78.21%), Netflix (49.64%), and TikTok (33.41%) (51).

## Conclusions

The functionalisation of play in the context of health promotion basically attempts to resolve a paradox: to integrate the motivational characteristics of free play into an actually unfree setting. This idea is well-intentioned and principally aimed at promoting the best interests of children. However, so far there has been no empirical evidence on its actual outcome. In a highly digitalized social world characterized by functionalization, isolation, and commercialization, there are hardly any spaces left in which children can have development-promoting experience without adult guidance. Serious games that are designed to promote a healthier behavior in children are therefore not necessarily the best answer on global health challenges. This is even more true when considering the potential opposite effects to those intended, namely if serious games increase the children's sedentary time. From a critical-sociological perspective, there is another interesting dynamic in the functionalization of play in form of e-games. Implicitly, this dynamic means that public health authorities delegate some of their responsibility for the promotion of citizens' health (comparable with similar tendencies in the field of education) to commercial enterprises whose interests by no means fully coincide with the tasks of public institutions. One could therefore say that governments do not fulfill their obligation to secure collective goods for society but that they leave gaps to be filled by commercial enterprises.

The question remains what public health strategies can be recommended to promote “healthy and development-promoting” play? Instead of pre-structured games, some of which take place on the computer, public health should rather aim to create stimulating movement environments in which children can act freely, invent their own rules, test these rules, measure each other, learn to cooperate, i.e., to make use of the socialization potential of sport and movement, which is neglected in guided, controlled worlds. This includes, firstly, the appropriation and questioning of norms and, secondly, the ability to develop design possibilities where social norms are lacking. Secondly, this includes learning the ability to act autonomously and to apply social norms in a reflective and flexible manner, both of which are a prerequisite for the development of an “ego-strength.” Thirdly, this also includes developing the competence to weigh self-interest against group interests in less pre-structured game worlds and to be able to make decisions that meet social obligations without neglecting one's own goals. PA-promoting free play areas should ideally be linked locally to schools and kindergartens to ensure that all children are reached. These areas should not be supervised and it should be left to the children themselves how and what they play. If one considers the socially contagious effects of free play, then it is to be expected that children will get moving.

Monitoring children's play then could focus on the infrastructures of settings in which free play should take place. Thereby, the design for the settings should also make sure to differentiate target groups in alignment with how they are affected from the decline of PA and to which degrees.

Parents who are involved in children's life, public health professionals, and researchers who are interested in understanding and monitoring children's play can bring back happiness to children's play. In this regard, children's play need to be contextualized in a holistic way. Parents, professionals, and researchers need to put children's perspectives and experience at center. From this perspective, the monitoring of play environments for children should primarily focus on whether play can “simply make children happy” (45). If this works, one can ultimately also expect benefits with regard to the children's physical, mental, and social health.

## Data Availability Statement

The original contributions presented in the study are included in the article/supplementary material, further inquiries can be directed to the corresponding author/s.

## Author Contributions

AF had the idea for the paper. AF and AT wrote and edited the draft article. All authors contributed to the article and approved the submitted version.

## Conflict of Interest

The authors declare that the research was conducted in the absence of any commercial or financial relationships that could be construed as a potential conflict of interest.
